# Constitutive Signaling by the Human Cytomegalovirus G Protein Coupled Receptor Homologs US28 and UL33 Enables Trophoblast Migration In Vitro

**DOI:** 10.3390/v14020391

**Published:** 2022-02-14

**Authors:** Nicholas Davis-Poynter, Helen E. Farrell

**Affiliations:** 1 School of Chemistry and Molecular Biosciences, The University of Queensland, St. Lucia 4072, Australia; nickdp.teach@gmail.com; 2 Centre for Child Health Research, The University of Queensland, Brisbane 4000, Australia

**Keywords:** cytomegalovirus, viral GPCR, trophoblast migration

## Abstract

Human cytomegalovirus (HCMV) encodes four homologs of G protein coupled receptors (vGPCRs), of which two, designated UL33 and US28, signal constitutively. UL33 and US28 are also conserved with chemokine receptors: US28 binds numerous chemokine classes, including the membrane bound chemokine, fractalkine; whereas UL33 remains an orphan receptor. There is emerging data that UL33 and US28 each contribute to HCMV associated disease, although no studies to date have reported their potential contribution to aberrant placental physiology that has been detected with HCMV congenital infection. We investigated the signaling repertoire of UL33 and US28 and their potential to enable trophoblast mobilization in vitro. Results demonstrate the constitutive activation of CREB by each vGPCR in ACIM-88 and HTR-8SVneo trophoblasts; constitutive NF-kB activation was detected for US28 only. Constitutive signaling by each vGPCR enabled trophoblast migration. For US28, fractalkine exhibited inverse agonist activity and dampened trophoblast migration. UL33 stimulated expression of both p38 mitogen activated (MAP) and Jun N-terminal (JNK) kinases; while p38 MAP kinase stimulated CREB, JNK was inhibitory, suggesting that UL33 dependent CREB activation was regulated by p38/JNK crosstalk. Given that chemokines and their receptors are important for placental development, these data point to the potential of HCMV UL33 and US28 to interfere with trophoblast responses which are important for normal placental development.

## 1. Introduction

Herpesvirus genomes show evidence of host gene capture events that have evolved to facilitate key aspects of the virus lifecycle. Homologues of G protein coupled receptors (GPCRs), the largest and most diverse membrane receptors in eukaryotes, have been identified in both beta- and gamma-herpesviridae [1,2,3,4] A number of the viral GPCRs (vGPCRs) show homology to chemokine receptors and exhibit constitutive G protein dependent signaling [4]. Their ability to “hijack” intracellular signaling cascades has been correlated to herpesvirus associated pathologies [5,6,7] Consequently, the viral GPCRs are antiviral targets [8,9].

The prototypes of the betaherpesvirus GPCRs were initially identified in human cytomegalovirus (HCMV; [10]). HCMV encodes four GPCRs, designated UL33, UL78, US27 and US28. Although none are essential for virus replication in vitro, their capture signifies importance for the virus lifecycle in vivo [11,12,13]. US27, US28 and UL33 are homologous to chemokine receptors and studies to date suggest they may subvert normal host–chemokine interactions [14]. In addition, UL33 and US28 signal constitutively [15], and, coupled with their incorporation into the mature virion envelope, possess the potential for modulating cellular signaling prior to de novo viral protein synthesis [16,17]. While UL33 is an orphan receptor, US28 constitutive signaling is further modified by a broad spectrum of host chemokines [18,19,20]. The impact of US28 and UL33 on the function of cells relevant to HCMV associated pathologies have provided valuable insight into their potential in vivo roles [21,22,23,24,25].

HCMV is the most common infectious cause of congenital disease, causing long term neurological defects in the developing fetus [26]. In addition to direct cytopathic damage, poor pregnancy outcomes may also be caused by HCMV associated pathological changes in the placenta [27]. Elegant immunohistochemical examination of placentae recovered from congenital HCMV infections demonstrated focal lytic infection of cytotrophoblasts, although virus antigens could be detected in other cell types in the absence of immediate-early (IE) expression [28,29,30]. Ex vivo infection of placental cultures with cell free HCMV revealed a broader range of infected cells [31]. Placental cells taken from cases of congenital infection demonstrate marked changes in gene expression compared with cells from uninfected counterparts, in particular genes associated with tissue angiogenesis, tissue remodeling, and inflammation [32,33,34,35]. Examination of HCMV infected trophoblasts has also revealed altered compositions in their extracellular matrix, aberrant migration and altered invasive potential compared with uninfected cells [36,37]. Given that chemokines/cytokines regulate these processes, it has been suggested that HCMV interferes with these communication networks [29,38,39].

Taken together, HCMV infected placentas exhibit altered trophoblast properties that are critical for establishing and maintaining a viable pregnancy. A number of these biological processes are underpinned by GPCR dependent mechanisms. Early studies of HCMV encoded GPCRs US28 and UL33 identified their constitutive signaling properties via heterologous expression in the well-established mammalian cell lines, HEK293 and COS-7 that possess numerous adenylyl cyclase, protein kinases A/C and G protein isoforms, making them an attractive readout to identify GPCR coupled networks [40]. Subsequent studies have been conducted in cells or tissues of relevance to herpesvirus infection, revealing viral GPCR (vGPCR) signaling characteristics that have the potential to contribute to pathological outcomes (reviewed by [5]). In these studies, we sought to evaluate the ability of HCMV US28 and UL33 to produce quantifiable, constitutive signaling in trophoblasts and to evaluate their contribution to trophoblast mobilization.

## 2. Materials and Methods

### 2.1. Cell Lines

Human embryonic kidney, (HEK293) cells (ATCC CRL-1573) were grown in Dulbecco’s modified Eagle’s medium (DMEM)-GlutaMAX (Invitrogen, Waltham, MA, USA) supplemented with 10% heat inactivated fetal calf serum (FCS), 180 U/mL penicillin and 45 μg/mL streptomycin at 37 °C and 5% CO_2_. Two trophoblast cell lines—the human choriocarcinoma–primary trophoblast hybrid ACIM-88 and the extravillous trophoblast HTR-8SVneo trophoblasts derived by immortalization of a human first trimester placenta [41]—were kindly provided by Dr Lois Salamonsen (Monash University, Melbourne, Australia). Trophoblast cell lines were grown in DMEM/F12 medium containing 10% charcoal stripped (cs)FCS, 180 U/mL penicillin and 45 μg/mL streptomycin at 37 °C and 5% CO_2_. We used ACIM-88 cells for most signaling studies reported here. To determine the response of US28 transfected trophoblasts to fractalkine, we used HTR-8Svneo cells, which lack detectable expression of the cellular fractalkine receptor [42].

### 2.2. Receptor Constructs, Chemokines, MAP Kinase Inhibitors

In this study, pcDNA3 US28 and UL33 expression constructs were either untagged or tagged at the C-terminus with EGFP. As UL33 is spliced [43], the cDNA was cloned for expression and C terminal tagging. Single point mutations of the transmembrane III “DRY” motif (DRY → DQY or DRY → NRY introduced by SOE-PCR have been described previously [41]. Deletion of 15 amino acids at the US28 N-terminus (ΔN[2-16]), which removed the fractalkine binding site, has also been described [44]. Furthermore, pcDNA3 expressing EGFP was used as a control for background level signaling and for evaluating transfection efficiency, except for bioplex experiments, for which pcDNA3 alone was used. The integrity of all constructs was confirmed by sequencing. The extracellular chemokine domain of human fractalkine was purchased from Peprotech, Australia. The p38, ERK1/2 and JNK MAP kinase inhibitors (SP292190, SP600125, PD98059, respectively) were purchased from Tocris Bioscience, UK.

### 2.3. Fluorescent Imaging Studies

Trophoblasts were transiently transfected with plasmids expressing US28-EGFP or UL33-EGFP (or their mutated derivatives) according to our previously described method [45]. Briefly, glass coverslip trophoblast cultures of transfected cells were washed twice with Hanks Balanced Salt Solution (HBSS; containing CaCl_2_ and MgCl_2_) and then incubated (for 10 min at 37 °C) with Image-iT LIVE plasma membrane (Invitrogen, Mt. Waverley, Australia), using 200 μL of labeling solution (5 μg/mL Alexa Fluor^594^ wheat germ agglutinin, WGA) per coverslip. Cells were then washed twice with HBSS and fixed using 2% paraformaldehyde-HBSS for 15 min at 37 °C. Fixed cells were washed three times with HBSS, and coverslips were mounted onto glass slides using ProLong Gold Antifade reagent (Invitrogen, Australia). Coverslips were examined using a Leica confocal software employing a zoom factor of ×2.5. Images were imported into Adobe Photoshop CS2, version 9, and overlays of the GFP and Alexa Fluor 594 images were produced.

### 2.4. ELISA to Measure vGPCR Expression

Cultures of HEK293 cells (5 × 10^4^/well) were transfected with either EGFP tagged US28 or UL33 plasmids at the indicated doses (in quadruplicate). The following day, cells were fixed and permeabilized. Expression of EGFP tagged receptors was quantified using anti-EGFP (Invitrogen, Mt. Waverley, Australia) and a goat anti-rabbit alkaline phosphatase conjugated antibody (Thermofisher, Victoria, Australia). Absorbance was measured at 405 nm (Bio-Tek/Agilent, Santa Clara, CA, USA).

### 2.5. Luciferase Reporter Assays

HEK293, ACIM-88 or HTR-8SVneo cells were seeded (5 × 10^4^ cells/well) in 96-well black and white isoplates (PerkinElmer Life and Analytical Sciences, Waltham, MA, USA) using their specific 10% FCS or csFCS growth medium (described above), but without antibiotics. One day after seeding, cells were co-transfected with various concentrations of pcDNA3 based receptor plasmid (0.6–5.0 μg) or the pcDNA3-EGFP control plasmid, and either 50 ng of pNFAT-Luc (Pathdetect cis-reporting system) or 50 ng of pFR-Luc and 6 ng of pFA2-CREB (Pathdetect trans-reporting system; both from Stratagene, San Diego, CA, USA) for the detection of NF-kB or CREB activation, respectively. Transfections (in quadruplicate) were carried out using OptiMEM and Lipofectamine 2000 reagent according to the manufacturer’s guidelines (Invitrogen). Serum free medium for ACIM-88 trophoblasts was also supplemented with transferrin (10 μg/mL), sodium selenite (25 ng/mL), linoleic acid (4.7 μg/mL), bovine serum albumin (BSA) and insulin (all from Merk). The medium was replaced with either DMEM-GlutaMAX/0.5% heat inactive FCS (HEK293 cells) or 0.5% heat inactivated csFCS (trophoblasts) without antibiotics at 5 h post-transfection. After a 24-h incubation, the cells were washed with phosphate buffered saline (PBS), and an equal volume of LucLite was added (PerkinElmer, Waltham, MA, USA). Luminescence was measured for 5 s at 22 °C, following 10 min of dark adaptation on a TopCount microplate scintillation and luminescence counter.

### 2.6. Bio-Plex Assay

HTR-8SVneo cells (6 × 10^5^ cells) were transfected (triplicate samples) with either 1 μg pcDNA3 or untagged US28 or UL33 plasmids. After 24 h, cells were lysed and incubated with antibody–biotin coupled beads specific for JNK, p38, ERK1/2, STAT3 and STAT5, according to the manufacturer’s instructions (Bio-Rad Laboratories, Hercules, CA, USA). After washing away the unbound biotinylated antibodies, the beads were incubated with a reporter streptavidin-phycoerythrin (SA-PE) conjugate and fluorescence of bead populations read on a Luminescence plate reader (BMG Labtech, Lorne, Australia). Positive and negative controls (Bio-Rad Laboratories, Hercules, CA, USA) were included for each analyte examined. Results are expressed relative to pcDNA3 signals.

### 2.7. Transwell Migration Assay

Transiently transfected HTR-8SVneo cells were serum starved for 18–24 h in DMEM/F12 medium. Cells were harvested with 0.05% trypsin (Merck, Australia) and trypsin activity was then quenched by the addition of DMEM/F12 with 2% cs-FCS. After two washes with PBS, cells were resuspended in serum free DMEM/F12 medium. A total of 20,000 cells were added to the upper chamber (duplicate or triplicate samples) of a transwell 8 um multiplate (Merck, Australia). The lower chamber contained the same medium, but with the addition of 10% csFCS. Where required, recombinant human fractalkine was added to wells at the indicated concentrations. The chamber was incubated for 22 h at 37 °C in a CO_2_ incubator to allow migration through the membrane. Cells remaining in the upper chamber were removed with a cotton swab and migration through the membrane was assessed by detaching cells on the upper surface of the membrane with a cotton swab. The membrane was then rinsed in HBSS before fixing in 100% methanol and air drying. Cells were stained with 5% Giemsa solution diluted in water for 10 min, rinsed briefly with water and air dried. Cells were visualized at ×40 magnification.

### 2.8. Statistical Analysis

For NF-kB and CREB signaling assays, vGPCRs were tested alongside the pcDNA3-EGFP control in quadruplicate. Assays were repeated 3 times, giving 12 sets of data for each receptor at each dose input. Raw data were normalized to the baseline pcDNA3-EGFP control (100 × S/C, where S is the value of the sample and C is the mean value of the vector control samples). For determining the effect of MAP kinase inhibitors, the same approach was used, except raw data for each vGPCR were normalized to the average signaling of the respective vGPCR in the absence of any inhibitors. For bioplex assays, all analyses were performed on triplicate biological samples; experiments were repeated three times. For transwell assays, migrated cells were counted from a total of 8–15 random fields across duplicate or triplicate biological samples. Migration assays were repeated 3–5 times. Comparisons were made using 1-way ANOVA with either Dunnett’s or Tukey’s post-tests (described in each figure legend) and were performed using GraphPad Prism, version 9. Differences were considered significant at >95% confidence.

## 3. Results

### 3.1. Expression of Wild Type and Mutated HCMV US28 and UL33 Constructs

**Figure 1:**
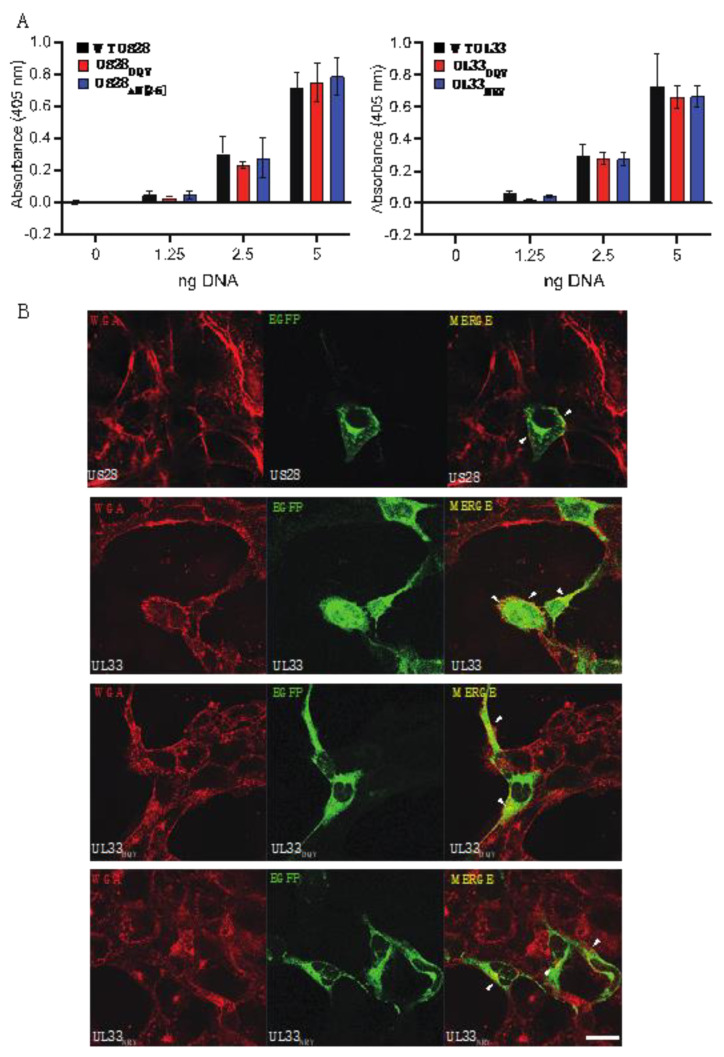
(**A**). Total protein expression of EGFP-tagged US28, UL33 and mutated derivatives in transfected HEK cells was determined by ELISA at the indicated plasmid doses. (**B**). ACIM-88 trophoblasts were transiently transfected with plasmids expressing EGFP-tagged UL33 or US28 or with mutated derivatives of UL33. Fluorescence was visualised by reactivity of Alexa-fluor 594-conjugated wheat germ aggluntinin (WGA; left panel). The distribution of the vGCPR by EGFP fluorescence is shown in the middle panel and the merged images in the right panels. Colocalization is shown by the arrows. Scale bar is shown at the bottom right and denotes 10 μM.

EGFP tagged US28 and UL33 were expressed in pcDNA3 [44,46]. The total protein expression of wild type (WT) and mutated derivatives of US28 and UL33 was evaluated in transfected HEK293 cells by ELISA (Figure 1A). Results showed that each mutation to the vGPCR had no intrinsic effect on protein expression. For US28, our previous studies demonstrated cell surface expression by detecting N terminally tagged WT and mutants, and by the ability (or not) to bind the cognate ligand, fractalkine. However, for UL33, numerous attempts to tag UL33 at the N terminus resulted in aberrant cellular expression (N. Davis-Poynter and H. Farrell, unpublished data). Moreover, as UL33 is an orphan receptor, ligand binding studies were not possible. Here, we investigated the cell surface expression of UL33 and mutated derivatives in ACIM-88 trophoblasts by confocal microscopy, using wheat germ agglutinin (WGA)^594^ as a cell surface marker (Figure 1B, left panel). Wild type US28 expression was also examined. EGFP tagged WT UL33 and US28 showed a similar intracellular expression, localized to perinuclear/Golgi and cytoplasmic vesicular structures (middle panel). We also detected their colocalization with WGA (filled arrows, right panel): prominent for UL33 and sparse for US28, possibly reflecting the high constitutive endocytosis of US28 at steady state [47]. Notably, we observed cell surface expression by both UL33 “DRY motif” point mutants.

### 3.2. UL33 and US28 Signal Constitutively in Trophoblasts

**Figure 2:**
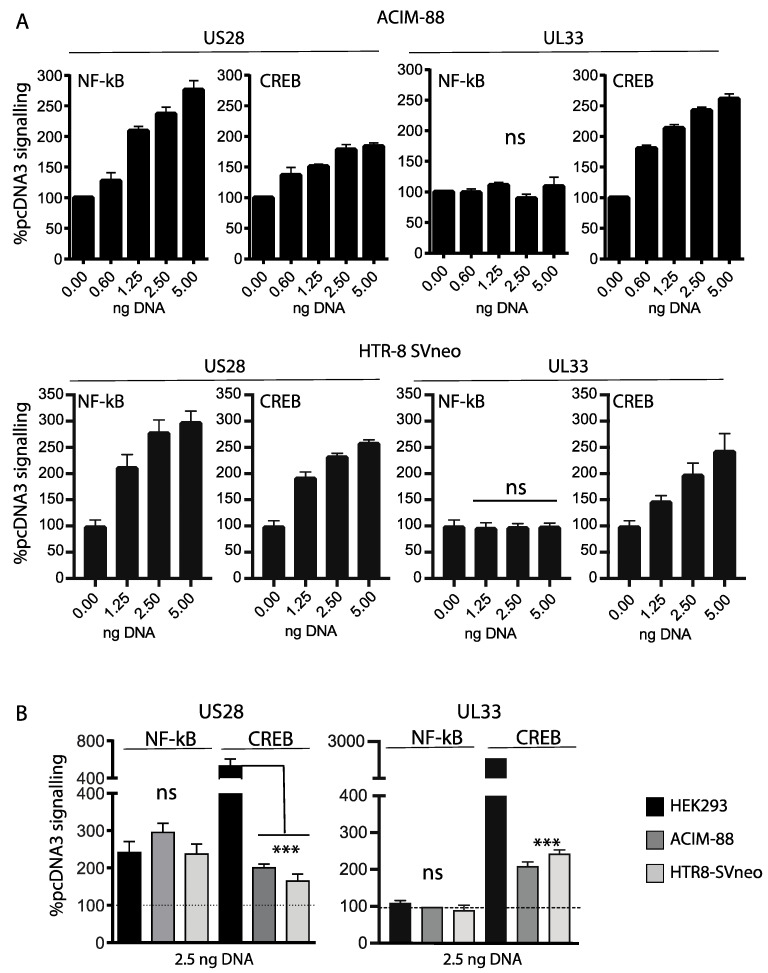
(**A**). Dose-dependent responses of ACIM-88 (upper panel) and HTR-8SVneo trophoblasts (lower panel) transiently transfected with either pcDNA3-EGFP, pcDNA3-US28 or pcDNA3-UL33 and the relevant CREB/NF-kB luciferase reporters. Signaling values are normalized against dose-dependent responses obtained with pcDNA3 alone. Comparisons of vGPCR transfected versus untransfected controls were made using 1-way ANOVA with Dunnett’s post-test. UL33 showed no evidence of NF-kB signaling at any dose, ns = not significant. US28 NF-kB/CREB and UL33 CREB signaling was significantly increased at all doses tested (*p* < 0.0001; not annotated on the figure to aid clarity) compared with untransfected controls. (**B**). Comparison of signaling amplitude between trophoblast lines and HEK293 cells following transient transfection with either US28 or UL33 (all at 2.5 ng), assessed using 1-way ANOVA with Tukey’s post-test. Signaling values are normalized against baseline signaling by pcDNA3 at the same dose; the dashed horizontal bar shows baseline activation levels. No differences were detected in the amplitude of NF-kB activation between cell lines; ns = not significant. ACIM-88 and HTR-8SVneo trophoblasts exhibited markedly reduced levels of CREB activation following US28 or UL33 transfection compared with HEK293 cell counterparts. *** = *p* < 0.001.

Constitutive GPCR signaling is revealed through the activation of second messengers in a gene dose dependent manner. Previous studies in HEK293 and COS-7 cells have demonstrated the constitutive activation of multiple pathways by UL33 and US28 [15]. Figure 2A shows dose response CREB and NF-Kb signaling in ACIM-88 and HTR-8SVneo trophoblasts (upper and lower panel, respectively); results are expressed relative to signaling detected at equivalent doses of pcDNA3-EGFP. Both UL33 and US28 constitutively activated CREB in ACIM-88 and HTR-8SVneo cells, whereas NF-kB activation was restricted to US28. When compared to signaling in HEK293 cells, the amplitudes of the NF-kB responses were equivalent for US28 and not detected for UL33 (Figure 2B). In contrast, both trophoblast cells gave a lower readout for CREB activation compared with HEK293 counterparts.

### 3.3. Contribution of MAP Kinases to US28- and UL33 Activation of Cellular Transcription Factors

**Figure 3:**
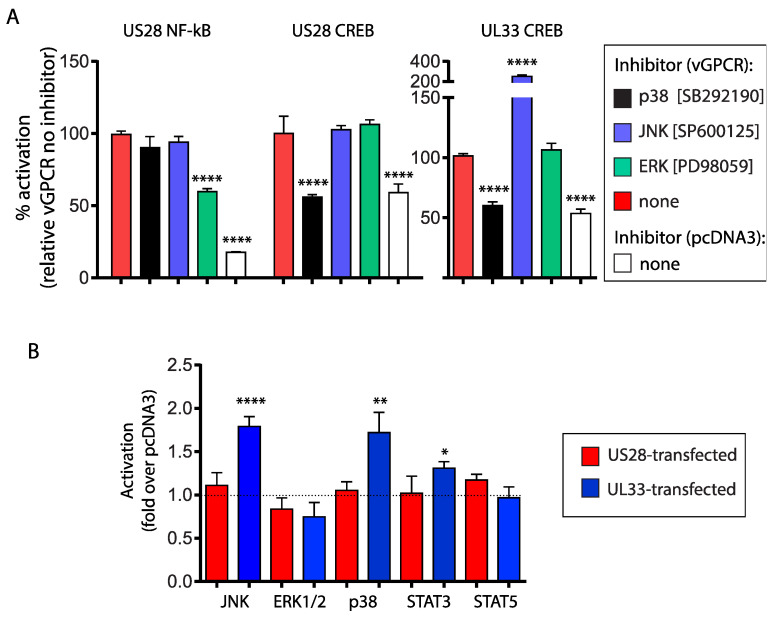
**(A**). ACIM-88 trophoblasts were transfected with 2.5 ng of either US28 or UL33; controls were transfected at the same dose with pcDNA3 vector. Immediately following transfection, cells were incubated with 5 µM of the p38 MAP kinase inhibitor SB292190 (black bars), 5 µM of the ERK1/2 inhibitor PD98059 (green bars) or 10 µM of the JNK inhibitor SP600125 (blue bars). Data are expressed as the % signalling of each vGPCR in the absence of any inhibitor (red bars). There was no effect of any inhibitor on pcDNA3-EGFP signaling (not shown); the level of signaling in pcDNA3-EGFP transfected cells in the absence of any inhibitor is shown (white bars). Comparisons between signaling detected in the presence and absence of inhibitors were made using 1-way ANOVA using Dunnett’s post-test. **** = *p* < 0.0001. (**B**). HTR-8SVneo cells were transfected with US28 or UL33 and were assessed 24 h later for upregulation of JNK, p38, STAT3 and STAT5 proteins using Bio-Plex immunoassay. Control cultures were transfected with pcDNA3 only. Results are expressed as the fold-activation of test samples over pcDNA3 controls (represented by the dashed line). Comparisons of UL33 and US28 transfected cells with their respective pcDNA3 controls were made using 1-way ANOVA with Dunnett’s post-test. **** = *p* < 0.0001; ** = *p* < 0.01, * = *p* < 0.05.

Given that the functions of transcriptional transactivators are regulated by upstream phosphorylation events, we investigated if MAP kinases ERK, JNK and p38 contributed to US28 and UL33 dependent, NF-kB and/or CREB activation. We exposed US28 and UL33 transfected ACIM-88 trophoblasts to specific MAP kinase inhibitors and measured their impact on signaling, compared with similar treatments on pcDNA3-EGFP transfected cells. The results are depicted in Figure 3A; data are presented relative to the signaling of each vGPCR in the absence of inhibitors (arbitrarily set at 100%; red bars). At the MAP kinase inhibitor doses used, we found they had no impact on baseline pcDNA3-EGFP NF-kB and/or CREB activation (data not shown; signaling indicated in the absence of inhibitors only). The application of the selective p38 MAP kinase inhibitor (SB292190; black bars) decreased CREB signaling mediated by UL33 and US28 to baseline levels. The dependence of p38 MAP kinase in US28 and UL33 CREB activation is consistent with the results of previous studies on COS-7 cells [15]. In contrast, the ERK inhibitor (PD98059; green bars), targeted US28 dependent NF-kB, but did not modulate CREB activation by either vGPCR. The inhibitory effect of ERK on NF-kB was incomplete since it did not reduce signaling to baseline pcDNA3 levels. We employed higher concentrations of PD98059 to investigate if it further reduced US28 NF-kB activation; however, they proved cytotoxic (data not shown).

While US28 CREB activation was not dependent on JNK (SP600125; blue bars), it markedly augmented UL33 dependent CREB activation, suggesting that JNK plays an inhibitory role in this pathway. To determine if JNK is differentially upregulated in UL33 transfected cells, we compared JNK levels in lysates HTR-8SVneo trophoblasts transfected with either UL33, US28 or pcDNA3 using Luminex magnetic bead immunoassay. The assay was multiplexed with p38 and ERK1/2 MAP kinases as well as for the transcription factors, STAT3 and STAT5. Results show increased JNK and p38 MAP kinases in UL33 transfected trophoblasts; a modest, but significant, increase in STAT3 was also observed for UL33 (Figure 3B). Taken together, the results suggest that the upregulation of p38 and JNK by UL33 have opposing effects on downstream CREB activation in ACIM-88 trophoblasts.

Despite contributing, in part, to US28 dependent NF-kB activation, we could not detect elevated levels of activated ERK1/2 by bead immunoassay in US28 transfected trophoblasts. Unlike agonist–GPCR engagements that result in a transient elevation of activated MAPK, constitutive GPCR signaling establishes a new steady state level in transfected cells. While the bead immunoassay measures “live” levels of ERK1/2 in response to the constitutive vGPCR, the luciferase assay quantifies amplified downstream signaling following protracted exposure of transfected cells to inhibitors. Thus, the lack of apparent correlation between assays may reflect assay design and readout sensitivity. Based on results, the data suggest that ERK1/2 contributes in part to US28 dependent NF-kB activation.

### 3.4. Effect of Transmembrane III “DRY” Motif mutations on Constitutive Signaling

**Figure 4:**
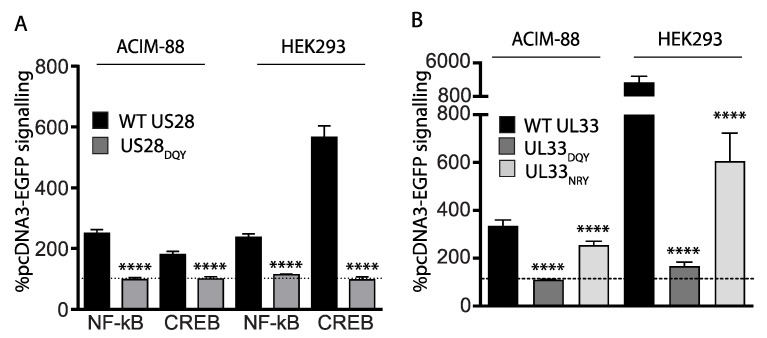
(**A**). Quantification of NF-kB and CREB signaling in ACIM-88 and HEK293 cells transfected with either wild type (WT) US28 or a DRY → DQY transmembrane III mutant. Signaling values are expressed relative to pcDNA3-EGFP controls at the same dose (indicated by the dashed horizontal line). Comparisons between WT US28 and the DQY derivative were made using Student’s 2-tailed *t*-test. **** = *p* < 0.0001. (**B**). CREB activation of WT UL33, UL33 DRY → DQY and UL33 DRY → NRY mutants in either ACIM-88 or HEK293 cells was quantified as for (**A**). Comparisons of the UL33 DQY/NRY mutants with WT UL33 were made using 1-way ANOVA with Dunnett’s post-test. **** = *p* < 0.0001.

The intracellular amino acid triplet motif (D/E)RY, found in transmembrane III is important for G protein engagement, although a number of vGPCR exhibit sequence diversity [48]. Of these residues, the arginine is the most highly conserved, and mutations of this residue generally ablate G protein mediated signaling. Both UL33 and US28 have prototypic “DRY” motifs. We prepared DRY → DQY expression counterparts for each of the viral GPCRs and compared their signaling with wild type counterparts in both ACIM-88 and in HEK cells. The DRY → DQY mutants showed a >95% reduction in CREB and NF-kB activation in both cell types (Figure 4A). Given that the viral chemokine receptor homologs possess a variation in the aspartate residue of the “DRY” motif and, indeed, the mouse CMV counterpart of UL33, (M33), possesses “NRY” rather than “DRY”, we also compared the signaling of a UL33 “NRY” mutant and found that, while signaling was partially preserved, the levels were nonetheless reduced significantly (Figure 4B).

### 3.5. Fractalkine Inhibits US28-Dependent NF-kB Activation

**Figure 5:**
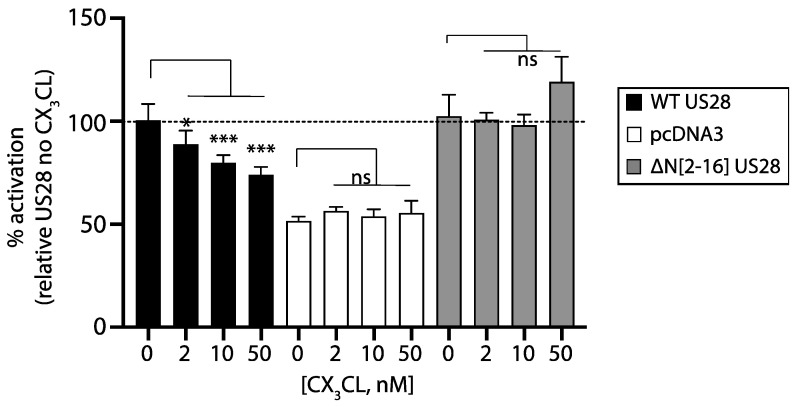
HTR-8SVneo trophoblasts were transfected with either US28, pcDNA3 or ∆N[1-6] US28 together with the NF-kB luciferase reporter. Transfected cells were incubated or not with fractalkine at the indicated concentrations. Results are presented normalized to US28 signaling in the absence of fractalkine (set at 100% and shown as a horizontal dashed line). Comparisons between fractalkine-treated and untreated controls were performed using 1-way ANOVA with Dunnett’s post-test. ns = not significant; * = *p* < 0.05; *** = *p* < 0.001.

While UL33 is an orphan receptor, numerous chemokines bind US28 [19,20]. Fractalkine binds US28 with high affinity and acts as an inverse agonist on NF-kB driven luciferase expression in COS-7 cells [49]. We investigated if fractalkine exhibited similar characteristics in US28 transfected trophoblasts. For these studies we used HTR-8SVneo cells, which, unlike ACIM-88 cells, do not express detectable levels of the fractalkine GPCR [42]. Control HTR-SVneo cells were transfected with an N terminal US28 mutant (ΔN[2-16]) that preserves constitutive NF-kB activation, but lacks the known binding sites for fractalkine [44]. To further control for the off-target effects of fractalkine, we transfected cells with pcDNA3. Results are expressed as the % signaling of WT US28 in the absence of fractalkine (Figure 5). We detected a dose dependent inhibition of NF-kB signaling by fractalkine in WT-US28 transfected cultures. In contrast, the N terminal US28 mutant signaled to equivalent levels as wild type US28 irrespective of fractalkine incubation. As expected, pcDNA3-EGFP failed to signal in the presence or absence of fractalkine. Thus, NF-kB inhibition by fractalkine was US28 specific.

### 3.6. Constitutive Signaling by US28 and UL33 Enables Trophoblast Migration

**Figure 6:**
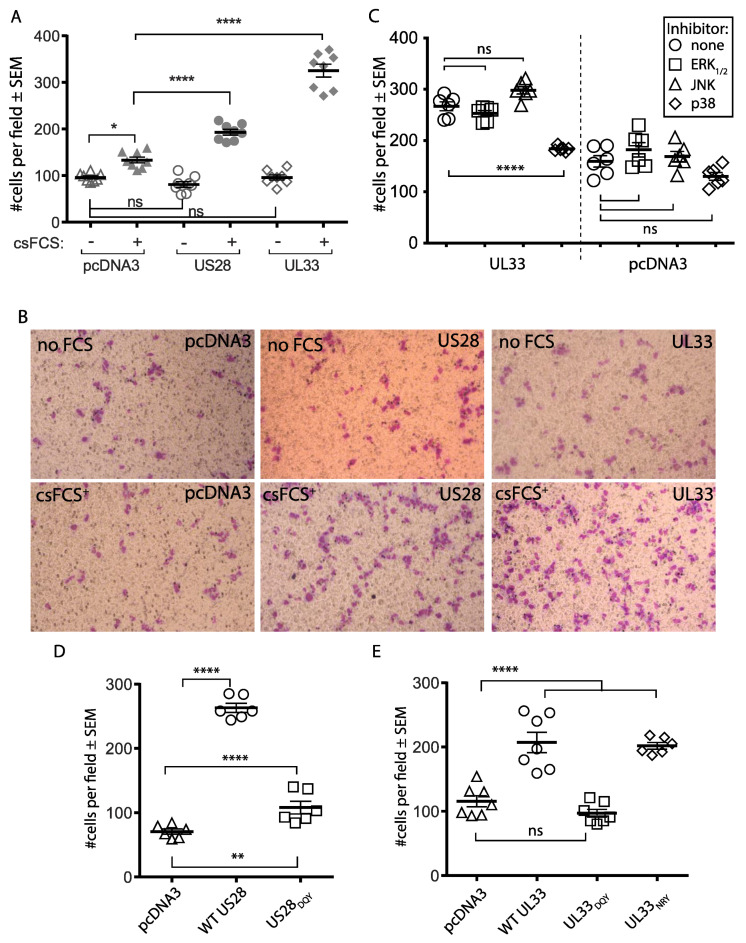
(**A**). HTR-8SVneo cells were transfected with either US28, UL33 or pcDNA3 and serum starved for 18–24 h. Cells were loaded onto the top chamber of 3 μM transwells, in the presence or absence of 10% csFCS in the lower chamber (in duplicate). The numbers of migrated cells on the underside of duplicate transwells were counted 24 h later (*n* = 8–9). Multiple comparisons between transfected and untransfected cells in the presence and absence of serum were made using 1-way ANOVA with Tukey’s post-test. Cells transfected with pcDNA3 showed a modest migration increase in the presence of csFCS (* = *p* < 0.05). Both US28 and UL33 each induced migration in the presence of csFCS that was significantly higher than pcDNA3 counterparts (**** = *p* < 0.0001). The data is representive of 5 experiments. (**B**). Examples of migrated HTR-8SVneo cells transfected with either pcDNA3 (left), US28 (middle) or UL33 (right) in the absence or presence of of csFCS (upper and lower panels respectively). (**C**). HTR-8SVneo cells transfected withUL33 or pcDNA3 as in (**A**) were treated or not with 5 μM ERK1/2, 10 μM JNK or 5 μM p38 MAPK inhibitors and migrated cells counted 24 h later. Multiple comparisons were made using 1-way ANOVA with Tukey’s post-test. The p38 MAPK inhibitor reduced UL33-dependent migration (**** = *p* < 0.0001). There was no effect of inhibitors on pcDNA3-transfected cells. ns = not significant. (**D**). HTR-8SVneo cells were transfected as in (**A**) with either WT US28 or US28DQY. Compared with WT US28, migration by US28DQY-transfected cells was signficantly reduced, but not fully ablated **** = *p* < 0.0001. ** = *p* < 0.01 E. HTR-8SVneo cells were transfected as in (**A**) with either WT UL33, UL33DQY or UL33NRY. The UL33DQY but not the UL33NRY ablated migration. ns = not significant, **** = *p* < 0.0001. Comparisons for each WT vGPCR with mutated derivatives in (**D**,**E**) were made using 1-way ANOVA with Dunnett’s post-test. The data shown in (**C**–**E**) are representive of 3 experiments.

We next investigated if the constitutive signaling of US28 and UL33 enabled trophoblast migration. We transfected HTR-8SVneo trophoblasts with either US28, UL33 or pcDNA3-EGFP. After 6 h, cells were washed and starved of serum overnight. A total of 20,000 cells in serum free medium were applied to the upper well of a transwell chamber. The lower well of the chamber contained the same medium, with or without the presence of 10% csFCS. After 16 h, cells that had migrated through the membrane were stained with Giemsa and counted. In the absence of csFCS in the lower chamber, there was no difference between transfectants in the number of migrated cells (Figure 6A). The addition of csFCS resulted in an increase in the migration of all transfectants, although both US28 and UL33 transfected cultures exhibited markedly increased numbers of migrated cells compared with pcDNA3-EGFP controls. Example fields of view comparing cultures lacking csFCS in the lower chamber with csFCS supplemented cultures are shown in (Figure 6B).

MAPK have been shown to be important regulators of cellular function, including cell proliferation, migration, differentiation, and death. Results presented in Figure 3B showed that UL33 transfected HTR-8SVneo trophoblasts expressed activated p38 and JNK MAPK. CREB activation was a downstream readout of their activity; while p38 MAPK upregulated CREB, JNK inhibited it. We sought to determine if inhibitors of p38 MAPK (SB292190), JNK (SP600125) or ERK1/2 (PD98059) impacted UL33 dependent migration in HTR-9SVneo trophoblasts (Figure 6C). The migration of control cultures transfected with pcDNA3 alone were unaffected by the MAPK inhibitors. Against the migration of UL33 transfected trophoblasts, the p38 MAPK inhibitor markedly reduced the migration of HTR-8SVneo cells (*p* < 0.0001), indicative of an important role in trophoblast mobilization. The application of the JNK inhibitor, which elevated UL33 dependent CREB signaling beyond that stimulated by UL33 alone, gave an upward trend in UL33 dependent migration, although this was not significantly higher than in its absence (*p* = 0.23).

Next, we compared the migration of HTR-8SVneo trophoblasts transfected with either WT US28 or UL33 with their DQY signaling null mutant counterparts. We also investigated if migration was enabled for the UL33 DRY → NRY mutant that possessed the partial preservation of CREB activation. Here, pcDNA3-EGFP provided a negative control. For both receptors, the DRY → DQY mutation reduced migration significantly. While US28_DQY_ transfected trophoblasts still exhibited minor enhancement above that of pcDNA3, the mutation ablated the UL33 dependent migratory activity (Figure 6D,E). In contrast, we found no impact of the UL33 DRY → NRY mutation on trophoblast migration. Taken together, the constitutive signaling of either US28 or UL33 transfected trophoblasts correlated with migration capability.

### 3.7. Fractalkine Restricts US28 Dependent Trophoblast Migration

**Figure 7:**
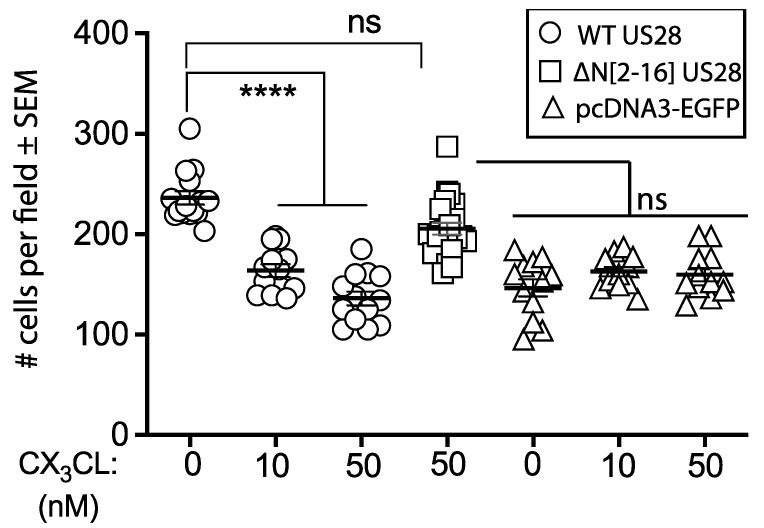
Fractalkine inhibits migration of HTR-8SVneo trophoblasts. HTR-8SVneo trophoblasts were transfected with either US28, pcDNA3 or US28 ΔN[1-6], serum starved for 24 h and loaded onto an upper transwell chamber. The lower chamber contained 10% csFCS, together with fractalkine at the indicated concentrations for 16 h. The number of cells on the lower interface of the transwells were counted across triplicate samples (*n* = 12–15). WT US28+ cultures in the presence of fractalkine showed significantly reduced migration compared with the absence of fractalkine. **** = *p* < 0.0001; ns = not significant. Fractalkine did not affect the migration of pcDNA3-EGFP or the US28 ΔN[1-6] mutant at any dose tested. (Only the highest fractalkine concentration is shown for ΔN[1-6] US28).

Given the positive correlation between constitutive signaling and trophoblast migration, we sought to determine if the known inverse agonist activity of human fractalkine on US28 impacted trophoblast migration capacity. We transfected HTR-8SVneo trophoblasts with either US28, ΔN[2-16] US28 or pcDNA3-EGFP and measured migration with or without the indicated concentration of fractalkine in the lower chamber. Results showed the dose dependent inhibition of migration for WT US28, but not for the ΔN[2-16] US28 counterpart, suggesting inhibition arose from cognate fractalkine–US28 interactions. There was no evidence of nonspecific inhibition by fractalkine on pcDNA-3 transfected HTR-8SVneo trophoblasts (Figure 7).

## 4. Discussion

Human cytomegalovirus (HCMV) is the leading viral cause of congenital infection, resulting in fetal and neonatal death and neurodevelopmental and sensorineural sequelae [26]. HCMV spreads systemically and placental infection is likely initiated via infected maternal blood myeloid cells [50]. Within the placenta, trophoblast invasion, migration and fusion are highly coordinated events that are regulated by macrophage interactions [51]. Studies of placentas taken from congenital infections demonstrate that HCMV markedly perturbs the signaling repertoire responsible for normal trophoblast function, correlating with their aberrant proliferation, migration, and invasion [27].

To date, most information relating to the capacity of CMV GPCRs in regulating cell migration has come from studies with US28. Previous studies have shown US28 to enable the migration of human arterial smooth muscle cells (SMC), making a case for its role in contributing to HCMV associated vascular disease, such as atherosclerosis [52]. Notably, US28 dependent migration varied between SMC types, pointing to US28 effects being cell type specific. The importance of the cellular microenvironment in the physiological readout of US28 activation was emphasized in studies that demonstrated that US28 dependent migration was modulated in a ligand specific manner: fractalkine inhibited RANTES signaling in SMC while RANTES inhibited fractalkine signaling in macrophages [21]. Similar cell type specific effects of vGPCR signaling on migration were reported for the MCMV UL33 homolog, M33 [53].

Little is known of the potential of the HCMV GPCRs to contribute to observed placental dysfunction. We report here that HCMV US28 and UL33 GPCRs exhibited constitutive G protein dependent signal transduction in ACIM-88 and HTR-8SVneo trophoblasts. As we were unable to quantify cell surface expression for UL33 and its mutated derivatives, the possibility exists that signaling as a result of UL33 mutations were the consequence of modified expression. However, given that the invariant arginine in the majority of GPCRs is critical for G protein engagement, it is unlikely that ablated signaling for UL33_DQY_ mutant resulted from global receptor loss from the cell surface. In addition, while reduced receptor levels may have accounted for reduced UL33NRY signaling, this mutation, nonetheless, had little significance on migration.

For each viral GPCR, gene dose dependent signaling was equivalent between the two trophoblast cell lines and their signaling repertoires were consistent with those reported for other cell lineages: NF-kB and CREB activated by US28, while UL33 targeted CREB only. Previous studies have identified NF-kB signaling for UL33 and its mouse CMV counterpart, M33 [15,54], but neither vGPCRs have exhibited this activity in our hands nor at similar doses in others [24], which may reflect differences between laboratories in the vGPCR variants or host cells used. A cognate US28 ligand, fractalkine, proved to be a potent inverse agonist to the constitutive NF-kB signaling of US28 in HTR-8SVneo trophoblasts, similar to the fractalkine modulation of US28 detected in some other cell lineages.

The MAP kinase family, represented by JNK, ERK and p38 MAPK, coordinate numerous signaling pathways upstream of transcription factors that regulate cellular homeostasis, including trophoblast differentiation and fusogenic activity, mobility and invasion [55]. Given that UL33 activated p38 and JNK MAP kinases in trophoblasts, the potential exists for HCMV to interfere with these downstream regulators. JNK did not appear to promote either CREB or NF-kb pathways; rather, in UL33 transfected trophoblasts, the application of the JNK inhibitor markedly enhanced CREB activation, suggesting JNK plays an inhibitory effect on CREB and antagonistic to p38 MAP kinase. Previous studies have revealed that JNK and p38 MAPK crosstalk as a key mechanism regulating cell proliferation, differentiation, invasion and survival, in the context of cancer development [56]. UL33 also activated STAT3 in HTR-8SVneo cells, consistent with findings in HEK293T cells and in glioblastoma U251 cells [57]. Similar to tumor cells, STAT3 is required for invasive properties in trophoblasts [58]. Taken together, the results provide evidence for the contribution of UL33 in proangiogenic and proliferative cellular responses.

The results of transwell migration experiments demonstrated that, for US28 and UL33, their independent constitutive signaling correlated with trophoblast mobilization. Firstly, for both receptors, the mutation of the invariant arginine of “DRY box” that ablated signaling, also reduced migration. Secondly, the exposure of a UL33 transfected trophoblast to the p38 MAP kinase inhibitor [SB292190], which was shown to ablate downstream constitutive CREB activation, also inhibited migration. Thirdly, fractalkine, a potent inverse agonist of US28, inhibited US28 dependent trophoblast migration.

Fractalkine exists in both membrane bound and soluble forms. Detected on the apical surface of syncytiotrophoblasts from early pregnancy, fractalkine may contribute to placental villi interactions with the circulating maternal blood [59]. For US28, fractalkine binding proved inhibitory for HTR-8SVneo cell migration at ligand concentrations that also correlated with its inverse agonist activity, suggesting the potential to modulate normal trophoblast responses. However, given the ability of US28 to bind numerous chemokines, coupled with the effect of membrane bound vs. soluble fractalkine on migration and signaling [60], it will be important in future studies to explore the functional impact of fractalkine–US28 interactions in different chemokine contexts and in multicellular systems that more closely resemble the in situ situation [61].

Trophoblast homeostasis is affected by numerous decidual factors, cytokines, chemokines and ligands of the EGFP and Wnt signaling pathways. Responses are temporally and spatially distributed and may be subject to autocrine and paracrine regulation [62]. WNT signaling contributes to the expansion of villous cytotrophoblast progenitors and extravillous trophoblast differentiation, and is important for normal placental morphogenesis and differentiation [63]. HCMV has been shown to disrupt both Wnt3a induced migration by interrupting canonical Wnt3a and noncanonical Wnt5a/JNK signaling to modify trophoblast migration [34,39]. The potential exists for the vGPCR to participate in these downstream signaling events.

Given their removal from whole tissue complexity, in vitro models can provide only a snapshot of HCMV vGPCR effects on trophoblast physiology. Transfection experiments performed here provided the means to detect a clean readout of single gene effects. The biological outcomes of signal regulators and transcriptional activators depend on both the intensity and duration of the individual signals produced and the extent of receptor cross-talk. Therefore, the results presented here are indicative of the dysregulatory potential for each viral GPCR; additional experiments are required to reveal the integrated physiological effects of the vGPCRs expressed by their natural promoters in the context of HCMV infection. In our hands, HTR-SVneo and ACIM-88 cells proved poorly permissive to infection with cell free HCMV strain Merlin, precluding such functional analyses. The poor permissiveness of cultured cell lines to infection does not necessarily negate readouts from transfection but highlights the need to explore infection models in primary cells, or 3D organoid cultures that mimic cell associated virus spread. Moreover, it is likely that multiple HCMV genes contribute to these trophoblast modifications, beyond vGPCR dependent mechanisms. Nevertheless, such “bottom up” studies provide clues to potential mechanisms that both facilitate HCMV traverse across the placental bottleneck to the developing fetus and pathological sequelae. As current HCMV antivirals are generally regarded as too toxic for use in pregnancy, there is a need for a new generation of antiviral therapies. In this respect, the GPCRs have an excellent track record as highly manipulable targets.

## Data Availability

Not applicable.
